# Supplementary material for: A Critical View of the Application of the APEX Software (Aqueous Photochemistry of Environmentally-Occurring Xenobiotics) to Predict Photoreaction Kinetics in Surface Freshwaters
Source: Molecules. 2019 Dec 18;25(1):9. doi: 10.3390/molecules25010009 (PMC7017383; doi:10.3390/molecules25010009)
Supplement: Supplementary file 1 [file molecules-25-00009-s001.zip › 3.2.4_gcc-4.4.0.zip!/3.2.4_gcc-4.4.0/3.2.4_gcc-4.4.0/doc/octave/html/interpreter/Concept-Index.html]

Concept Index - Untitled


Next: Function Index,
Previous: Copying,
Up: Top


---

## Concept Index

- #: Single Line Comments
- #!: Executable Octave Programs
- #{: Block Comments
- %: Single Line Comments
- %{: Block Comments
- `--braindead`: Command Line Options
- `--debug`: Command Line Options
- `--doc-cache-file` filename: Command Line Options
- `--echo-commands`: Command Line Options
- `--exec-path` path: Command Line Options
- `--help`: Command Line Options
- `--image-path` path: Command Line Options
- `--info-file` filename: Command Line Options
- `--info-program` program: Command Line Options
- `--interactive`: Command Line Options
- `--line-editing`: Command Line Options
- `--no-history`: Command Line Options
- `--no-init-file`: Command Line Options
- `--no-init-path`: Command Line Options
- `--no-line-editing`: Command Line Options
- `--no-site-file`: Command Line Options
- `--norc`: Command Line Options
- `--path` path: Command Line Options
- `--persist`: Command Line Options
- `--quiet`: Command Line Options
- `--silent`: Command Line Options
- `--traditional`: Command Line Options
- `--verbose`: Command Line Options
- `--version`: Command Line Options
- `-?`: Command Line Options
- `-d`: Command Line Options
- `-f`: Command Line Options
- `-H`: Command Line Options
- `-h`: Command Line Options
- `-i`: Command Line Options
- `-p` path: Command Line Options
- `-q`: Command Line Options
- `-v`: Command Line Options
- `-V`: Command Line Options
- `-x`: Command Line Options
- `...` continuation marker: The <code>try</code> Statement
- `.octaverc`: Startup Files
- `\` continuation marker: The <code>try</code> Statement
- acknowledgements: Acknowledgements
- addition: Arithmetic Ops
- and operator: Boolean Expressions
- anonymous functions: Function Handles Inline Functions and Anonymous Functions
- `ans`: Variables
- answers, incorrect: Bug Reporting
- answers, incorrect: Bug Criteria
- area series: Area series
- arguments in function call: Calling Functions
- arithmetic operators: Arithmetic Ops
- assignment expressions: Assignment Ops
- assignment operators: Assignment Ops
- axes graphics object: Graphics Objects
- axes properties: Axes Properties
- backends, graphics: Graphics backends
- bar series: Bar series
- batch processing: Executable Octave Programs
- block comments: Block Comments
- body of a loop: The <code>while</code> Statement
- boolean expressions: Boolean Expressions
- boolean operators: Boolean Expressions
- `break` statement: The <code>break</code> Statement
- bug criteria: Bug Criteria
- bug report mailing lists: Bug Lists
- bugs: Reporting Bugs
- bugs, investigating: Bug Reporting
- bugs, known: Trouble
- bugs, reporting: Bug Lists
- bugs, reporting: Bug Reporting
- built-in data types: Built-in Data Types
- built-in function: A Sample Function Description
- callbacks: Callbacks
- `case` statement: The <code>switch</code> Statement
- `catch`: The <code>try</code> Statement
- cell arrays: Cell Array Objects
- cell arrays: Cell Arrays
- character strings: Strings
- character strings: String Objects
- Cholesky factorization: Matrix Factorizations
- clearing the screen: Cursor Motion
- coding standards: Tips and Standards
- coding standards: Contributing Guidelines
- colors, graphics: Colors
- comma separated lists: Comma Separated Lists
- command and output logs: Diary and Echo Commands
- command completion: Commands For Completion
- command descriptions: A Sample Command Description
- command echoing: Diary and Echo Commands
- command history: Commands For History
- command options: Command Line Options
- command-line editing: Command Line Editing
- comments: Comments
- comparison expressions: Comparison Ops
- complex-conjugate transpose: Arithmetic Ops
- containers: Data Containers
- continuation lines: The <code>try</code> Statement
- `continue` statement: The <code>continue</code> Statement
- contour series: Contour groups
- contributing to Octave: How You Can Contribute to Octave
- contributors: Preface
- conversion specifications (`printf`): Formatted Output
- conversion specifications (`scanf`): Formatted Input
- copyright: Copying
- core dump: Bug Criteria
- cs-lists: Comma Separated Lists
- customizing `readline`: Customizing readline
- customizing the prompt: Customizing the Prompt
- DAE: Differential Equations
- data sources in object groups: Data sources in object groups
- data structures: Data Structures
- data structures: Data Structure Objects
- data types: Data Types
- data types, built-in: Built-in Data Types
- data types, user-defined: User-defined Data Types
- decrement operator: Assignment Ops
- default arguments: Default Arguments
- default graphics properties: Managing Default Properties
- defining functions: Functions and Scripts
- description format: Format of Descriptions
- diary of commands and output: Diary and Echo Commands
- Differential Equations: Differential Equations
- diffs, submitting: Sending Patches
- distribution of Octave: Distribution
- division: Arithmetic Ops
- `do-until` statement: The <code>do-until</code> Statement
- documentation fonts: Fonts
- documentation notation: Evaluation Notation
- documenting functions: Comments and the Help System
- documenting Octave programs: Comments
- documenting user scripts: Comments and the Help System
- Dulmage-Mendelsohn decomposition: Mathematical Considerations
- dynamic-linking: Dynamically Linked Functions
- echoing executing commands: Diary and Echo Commands
- editing the command line: Command Line Editing
- element-by-element evaluation: Element-by-element Boolean Operators
- `else` statement: The <code>if</code> Statement
- `elseif` statement: The <code>if</code> Statement
- Emacs TAGS files: Using Octave Mode
- `end` statement: Statements
- `end_try_catch`: The <code>try</code> Statement
- `end_unwind_protect`: The <code>unwind\_protect</code> Statement
- `endfor` statement: The <code>for</code> Statement
- `endfunction` statement: Defining Functions
- `endif` statement: The <code>if</code> Statement
- `endswitch` statement: The <code>switch</code> Statement
- `endwhile` statement: The <code>while</code> Statement
- equality operator: Comparison Ops
- equality, tests for: Comparison Ops
- equations, nonlinear: Nonlinear Equations
- erroneous messages: Bug Criteria
- erroneous results: Bug Reporting
- erroneous results: Bug Criteria
- error bar series: Error bar series
- error message notation: Error Messages
- error messages: Errors
- error messages, incorrect: Bug Criteria
- escape sequence notation: Escape Sequences in string constants
- evaluation notation: Evaluation Notation
- executable scripts: Executable Octave Programs
- execution speed: Coding Tips
- exiting octave: Running Octave
- exiting octave: Quitting Octave
- exponentiation: Arithmetic Ops
- expression, range: Ranges
- expressions: Expressions
- expressions, assignment: Assignment Ops
- expressions, boolean: Boolean Expressions
- expressions, comparison: Comparison Ops
- expressions, logical: Boolean Expressions
- factorial function: Recursion
- fatal signal: Bug Criteria
- figure graphics object: Graphics Objects
- figure properties: Figure Properties
- flag character (`printf`): Output Conversion Syntax
- flag character (`scanf`): Input Conversion Syntax
- flying high and fast: Variables
- `for` statement: The <code>for</code> Statement
- Fordyce, A. P.: Calling a Function by its Name
- Frobenius norm: Basic Matrix Functions
- function descriptions: A Sample Function Description
- function file: Function Files
- function file: A Sample Function Description
- `function` statement: Defining Functions
- functions, user-defined: Functions and Scripts
- funding Octave development: How You Can Contribute to Octave
- general p-norm: Basic Matrix Functions
- getting a good job: Variables
- `global` statement: Global Variables
- global variables: Global Variables
- gnuplot interaction: Interaction with gnuplot
- graphics: Plotting
- graphics backends: Graphics backends
- graphics colors: Colors
- graphics line styles: Line Styles
- graphics marker styles: Marker Styles
- graphics object properties: Graphics Object Properties
- graphics object, axes: Graphics Objects
- graphics object, figure: Graphics Objects
- graphics object, image: Graphics Objects
- graphics object, line: Graphics Objects
- graphics object, patch: Graphics Objects
- graphics object, root figure: Graphics Objects
- graphics object, surface: Graphics Objects
- graphics object, text: Graphics Objects
- graphics properties, default: Managing Default Properties
- greater than operator: Comparison Ops
- group objects: Quiver group
- group objects: Scatter group
- group objects: Stair group
- group objects: Surface group
- handle, function handles: Function Handles Inline Functions and Anonymous Functions
- header comments: Function Headers
- help, on-line: Getting Help
- help, user-defined functions: Comments and the Help System
- help, where to find: Service
- Hermitian operator: Arithmetic Ops
- Hessenberg decomposition: Matrix Factorizations
- history: Preface
- history of commands: Commands For History
- `if` statement: The <code>if</code> Statement
- image graphics object: Graphics Objects
- image properties: Image Properties
- improving Octave: Sending Patches
- improving Octave: Bug Criteria
- incorrect error messages: Bug Criteria
- incorrect output: Bug Criteria
- incorrect output: Bug Reporting
- incorrect results: Bug Reporting
- incorrect results: Bug Criteria
- increment operator: Assignment Ops
- infinity norm: Basic Matrix Functions
- initialization: Startup Files
- inline, inline functions: Function Handles Inline Functions and Anonymous Functions
- input conversions, for `scanf`: Table of Input Conversions
- input history: Commands For History
- installation trouble: Trouble
- installing Octave: Installation
- introduction: Introduction
- invalid input: Bug Criteria
- job hunting: Variables
- known causes of trouble: Trouble
- less than operator: Comparison Ops
- line graphics object: Graphics Objects
- line properties: Line Properties
- line series: Line series
- line styles, graphics: Line Styles
- loadable function: A Sample Function Description
- loading data: Simple File I/O
- logging commands and output: Diary and Echo Commands
- logical expressions: Boolean Expressions
- logical operators: Boolean Expressions
- loop: The <code>while</code> Statement
- looping over structure elements: Looping Over Structure Elements
- LP: Optimization
- LU decomposition: Iterative Techniques
- LU decomposition: Matrix Factorizations
- lvalue: Assignment Ops
- mapping function: A Sample Function Description
- marker styles, graphics: Marker Styles
- matching failure, in `scanf`: Formatted Input
- matrices: Matrices
- matrix multiplication: Arithmetic Ops
- maximum field width (`scanf`): Input Conversion Syntax
- messages, error: Errors
- mex: Mex-Files
- mex-files: Mex-Files
- minimum field width (`printf`): Output Conversion Syntax
- missing data: Missing Data
- mkoctfile: Oct-Files
- multi-line comments: Block Comments
- multiplication: Arithmetic Ops
- negation: Arithmetic Ops
- NLP: Optimization
- nonlinear equations: Nonlinear Equations
- nonlinear programming: Optimization
- not operator: Boolean Expressions
- numeric constant: Numeric Objects
- numeric constant: Numeric Data Types
- numeric value: Numeric Data Types
- numeric value: Numeric Objects
- object groups: Object Groups
- oct: Oct-Files
- oct-files: Oct-Files
- Octave command options: Command Line Options
- Octave development: Contributing Guidelines
- `octave-tags`: Using Octave Mode
- ODE: Differential Equations
- on-line help: Getting Help
- operator precedence: Operator Precedence
- operators, arithmetic: Arithmetic Ops
- operators, assignment: Assignment Ops
- operators, boolean: Boolean Expressions
- operators, decrement: Assignment Ops
- operators, increment: Assignment Ops
- operators, logical: Boolean Expressions
- operators, relational: Comparison Ops
- optimization: Optimization
- options, Octave command: Command Line Options
- or operator: Boolean Expressions
- oregonator: Ordinary Differential Equations
- `otherwise` statement: The <code>switch</code> Statement
- output conversions, for `printf`: Table of Output Conversions
- patch graphics object: Graphics Objects
- patch properties: Patch Properties
- patches, submitting: Sending Patches
- `persistent` statement: Persistent Variables
- persistent variables: Persistent Variables
- personal startup file: Startup Files
- plotting: Plotting
- precision (`printf`): Output Conversion Syntax
- printing notation: Printing Notation
- program, self contained: Executable Octave Programs
- project startup file: Startup Files
- prompt customization: Customizing the Prompt
- QP: Optimization
- QR factorization: Matrix Factorizations
- quadratic programming: Optimization
- quitting octave: Quitting Octave
- quitting octave: Running Octave
- quiver group: Quiver group
- quotient: Arithmetic Ops
- range expressions: Ranges
- `readline` customization: Customizing readline
- relational operators: Comparison Ops
- reporting bugs: Reporting Bugs
- reporting bugs: Bug Lists
- results, incorrect: Bug Reporting
- results, incorrect: Bug Criteria
- root figure graphics object: Graphics Objects
- saving data: Simple File I/O
- scatter group: Scatter group
- Schur decomposition: Matrix Factorizations
- script files: Functions and Scripts
- scripts: Executable Octave Programs
- self contained programs: Executable Octave Programs
- series objects: Line series
- series objects: Stem Series
- series objects: Area series
- series objects: Error bar series
- series objects: Contour groups
- series objects: Bar series
- short-circuit evaluation: Short-circuit Boolean Operators
- side effect: Assignment Ops
- singular value decomposition: Matrix Factorizations
- site startup file: Startup Files
- speedups: Coding Tips
- stair group: Stair group
- standards of coding style: Tips and Standards
- startup: Startup Files
- startup files: Startup Files
- statements: Statements
- stem series: Stem Series
- strings: Strings
- strings: String Objects
- Structural Rank: Sparse Linear Algebra
- structure elements, looping over: Looping Over Structure Elements
- structures: Data Structure Objects
- structures: Data Structures
- submitting diffs: Sending Patches
- submitting patches: Sending Patches
- subtraction: Arithmetic Ops
- suggestions: Bug Criteria
- surface graphics object: Graphics Objects
- surface group: Surface group
- surface properties: Surface Properties
- `switch` statement: The <code>switch</code> Statement
- TAGS: Using Octave Mode
- test functions: Test and Demo Functions
- tests for equality: Comparison Ops
- text graphics object: Graphics Objects
- text properties: Text Properties
- tips: Tips and Standards
- transpose: Arithmetic Ops
- transpose, complex-conjugate: Arithmetic Ops
- troubleshooting: Trouble
- `try` statement: The <code>try</code> Statement
- unary minus: Arithmetic Ops
- undefined behavior: Bug Criteria
- undefined function value: Bug Criteria
- `unwind_protect` statement: The <code>unwind\_protect</code> Statement
- `unwind_protect_cleanup`: The <code>unwind\_protect</code> Statement
- use of comments: Comments
- user-defined data types: User-defined Data Types
- user-defined functions: Functions and Scripts
- user-defined variables: Variables
- `varargin`: Variable-length Argument Lists
- `varargout`: Variable-length Return Lists
- variable descriptions: A Sample Variable Description
- variable-length argument lists: Variable-length Argument Lists
- variable-length return lists: Variable-length Return Lists
- variables, global: Global Variables
- variables, persistent: Persistent Variables
- variables, user-defined: Variables
- version startup file: Startup Files
- warranty: Copying
- `while` statement: The <code>while</code> Statement
- wrong answers: Bug Criteria
- wrong answers: Bug Reporting
- ~/.inputrc: Customizing readline
- `~/.octaverc`: Startup Files
